# A novel mutation in exon 9 of Cullin 3 gene contributes to aberrant splicing in pseudohypoaldosteronism type II

**DOI:** 10.1002/2211-5463.12389

**Published:** 2018-02-10

**Authors:** Leping Shao, Li Cui, Jingru Lu, Yanhua Lang, Irene Bottillo, Xiangzhong Zhao

**Affiliations:** ^1^ Department of Nephrology The Affiliated Hospital of Qingdao University Qingdao China; ^2^ Division of Medical Genetics Department of Molecular Medicine Sapienza University San Camillo‐Forlanini Hospital Rome Italy; ^3^ Central Laboratory The Affiliated Hospital of Qingdao University Qingdao China

**Keywords:** CUL3, exonic splicing enhancers, exonic splicing silencers, pseudohypoaldosteronism type II, splicing modulation

## Abstract

Pseudohypoaldosteronism type II (PHAII) is a rare renal tubular disease that is inherited in an autosomal dominant manner. Mutations in four genes (*WNK1*,*WNK4*,*CUL3,* and *KLHL3*) have been identified to be responsible for this disease. Cullin 3 (CUL3) and KLHL3 are subunits of Cullin–RING E3 ubiquitin ligase complexes, and the serine–threonine kinases WNK1 and WNK4 are substrates of this ubiquitin ligase. For *CUL3*, all mutations associated with PHAII exclusively lead to exon 9 skipping. In this study, we identified a Chinese PHAII kindred caused by a novel synonymous mutation (c.1221A > G p.Glu407Glu) in *CUL3*, and explored its effects on exon 9 abnormal splicing through an *in vitro* splicing assay and study of the patients’ RNA. We obtained evidence that this synonymous mutation leads to complete exon 9 skipping, and *in silico* bioinformatics analysis demonstrated that the *CUL3* c.1221A > G mutation might decrease the ratio of exonic splicing enhancers and silencers. This is the first report of PHAII in Chinese patients with a novel *CUL3* mutation. Our findings add a novel pathogenic splicing variant to the *CUL3* mutational spectrum and provide reference for further research on mechanisms of splicing modulation and development of potential therapeutic reagents for PHAII.

**Abbreviations**
DMD: Duchenne muscular dystrophy
ESEs: exonic splicing enhancers
ESSs: exonic splicing silencers
GFR: glomerular filtration rate
OSR1: oxidative stress responsive 1
PHAII: pseudohypoaldosteronism type II
SPAK: Ste20‐related proline–alanine‐rich kinase

Pseudohypoaldosteronism type II (PHAII), also known as Gordon syndrome or familial hyperkalemic hypertension, is a rare inherited disease featuring hypertension, hyperkalemia, hyperchloremic metabolic acidosis, normal glomerular filtration rate (GFR), and sensitivity to thiazide diuretics 1, 2. To date, mutations of four genes have been identified to be responsible for this disease 3, 4.

WNK1 and WNK4, belonging to the WNK (with‐no‐lysine kinase) family of serine–threonine kinases, are the first identified causal genes of PHAII 3. They constitute a phosphorylation signal cascade with Ste20‐related proline–alanine‐rich kinase (SPAK) or oxidative stress responsive 1 (OSR1), which target and regulate the activity of solute carrier family 12a (SLC12a) transporters, including thiazide‐sensitive NaCl cotransporter (NCC) and Na‐K‐Cl cotransporter (NKCC), to maintain the electrolyte homeostasis 5. Disease‐causing mutations in *WNK1* and *WNK4* lead to overactivation of NCC and impaired K^+^ secretion; this increases salt reabsorption and intravascular volume, causing hypertension and electrolyte disorder 6, 7.

The association of Kelch‐like 3 (*KLHL3*) and Cullin 3 (*CUL3*) genes with PHAII was recognized for the first time in 2012 4. As the core subunit, Cullin 3 assembles with KLHL3 to form Cullin–RING E3 ubiquitin ligase complexes, which mediates the substrates ubiquitination bound by KLHL3. Among them, WNK1 and WNK4 are the important substrates of Cullin–RING E3 ubiquitin ligase, once their ubiquitylation and degradation process is impaired due to pathogenic mutation in *CUL3* or *KLHL3*, which will lead to abnormal activation of WNK/OSR1/SPAK‐NCC signaling cascade in the kidney, thus breaking electrolyte homeostasis and eliciting PHAII phenotype 8, 9, 10, 11, 12, 13.

Regarding *CUL3* mutations, sixteen mutations have been described to be associated with PHAII so far 4, 14, 15 (Table S1). Of note, all these mutations distributed in sites involved in splicing of exon 9, such as intron 8 splice acceptor, intron 9 splice donor, putative intron 8 splice branch site, and putative exonic splicing enhancers (ESEs) and/or silencers (ESSs) in exon 9. Interestingly, all the described DNA changes exclusively lead to exon 9 skipping, producing an in‐frame fusion of exons 8 and 10, and a disabled protein lacking 57 amino acids in the region linking BTB‐binding and RING‐binding domains, which is essential for CUL3 function 3, 4.

Here, we report a novel synonymous mutation *CUL3* c.1221A > G (p.Glu407Glu) identified in a Chinese PHAII kindred including four affected patients. Furthermore, we confirmed that this mutation caused exon 9 skipping by an *in vitro* splicing assay and by studying the patients’ RNA. *In silico* analyses highlighted that the exon 9 skipping might be resulted from reduction in the ratio of ESEs to ESSs.

## Subjects and methods

### Patients and diagnostic criteria

**Figure 1:**
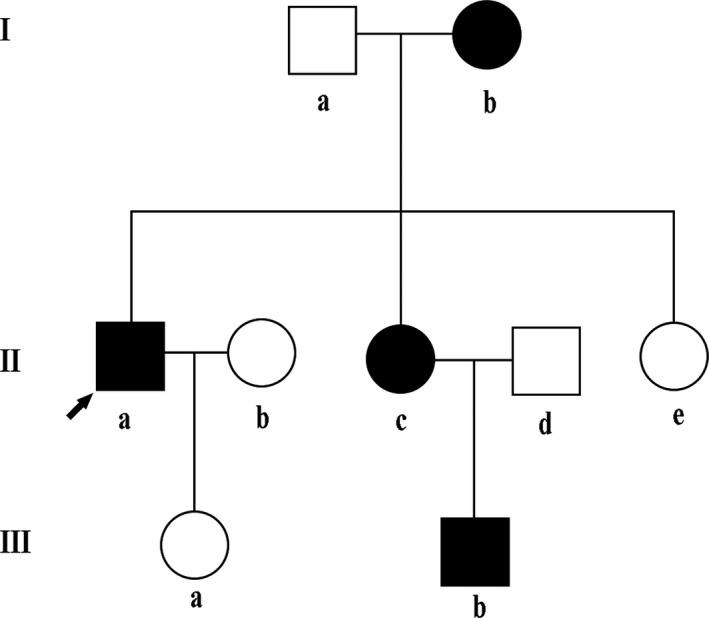
Pedigree of the Chinese family with pseudohypoaldosteronism type II. □, male ○, female; ■, male patient; ●, female patient; ↗, proband.

Patients with typical features of PHAII were enrolled for further confirmation based on the following diagnostic criteria 16, 17: (a) unknown cause of hypertension (blood pressure > 140/90 mmHg in adults and above the 95th percentile rank of age and sex in children and adolescents), (b) hyperkalemia (serum K ≥ 5.3 mmol·L^−1^), patients with hyperkalemia caused by drugs such as ACEI, ARB, or potassium‐sparing diuretics were excluded from this study, and (c) glomerular filtration rate > 90 mL·min^−1^·1,73 m^−2^. In this study, the subject was a Chinese kindred from Shandong province, China (Fig. 1). Four of nine family members were preliminarily diagnosed as PHAII according to their clinical characterizations and laboratory findings (Table 1). After written informed consent was obtained, blood samples were collected from all family members. The study protocol was approved by the Ethics Committee of the Affiliated Hospital of Qingdao University.

**Table 1:** Clinical features and biochemical data of four patients in the Chinese family with pseudohypoaldosteronism type II

Patients	Gender	Age (years)	Height (cm)	Systolic BP (mmHg)	Diastolic BP (mmHg)	SK (mmol·L^−1^)	Blood pH	SCl (mmol·L^−1^)	SHCO_3_ (mmol·L^−1^)	SCr (μmol·L^−1^)	Renin (ng·mL^−1^·h^−1^)^a^	Aldosterone (pg·mmL^−1^)^b^
Ib	Female	56	155 (159)^c^	175	120	6.1	7.250	110	16.0	80	0.05	23
IIa	Male	35	166 (170)^c^	160	105	6.5	7.227	113	14.2	94	0.08	78
IIc	Female	33	154 (159)^c^	165	110	6.3	7.272	109	16.3	87	0.03	11
IIIb	Male	12	142 (150)^c^	145	97	6.6	7.200	114	13.1	55	0.1	12

S, serum; Cr, creatinine; anormal range 0.1–2.9 ng·mL^−1^·h^−1^; bnormal range 29–161 pg·mL^−1^; cvalues in parentheses refer to the mean height values in Chinese adults and children.

### Genetic mutation analysis

Genomic DNA was extracted from the peripheral blood lymphocytes using Blood Genome DNA Extraction Kit (TaKaRa, Japan). For *WNK1*,* WNK4,* and *KLHL3* genes, all the coding regions and adjacent intronic segments were amplified according to previous studies 16, 17, 18. Primers for amplifying exon 9 of *CUL3* and its adjacent segments were used as follows: forward primer (5′‐TTACCTTATGCCTCCAAT‐3′) and reverse primer (5′‐CTCTGAATGTCCCTGAAC‐3′). The PCR amplification reaction was performed using EX Taq polymerase kit (Takara, Japan) according to the manufacturer's instructions. Then, PCR samples were subjected to bidirectional sequencing by Sangon Biotech Company (Shanghai). One hundred unrelated healthy subjects were selected as a control group. Sequence analysis and alignment were performed by biological software Chromas 2.31 and Vector NTI Advance 11.5. To analyze the potential effect of variants in the splice prediction, *in silico* analyses by HSF 3.0 software (http://www.umd.be/HSF3/HSF.shtml) were performed.

### Minigene construction and *in vitro* splicing analysis

To test the impact of mutation on splicing, an *in vitro* minigene splicing assay was performed using the pSPL3 exon‐trapping vector according to our previous reports 19, 20, 21. Briefly, fragments with the wild or mutant alleles containing exon 9 (171 bp), flanked by upstream intronic sequence (267 bp) and downstream intronic sequence (185 bp), were cloned into the splicing vector pSPL3 using specific primers (forward, 5′‐TTACCTTATGCCTCCAAT‐3′; reverse, 5′ ‐CTCTGAATGTCCCTGAAC‐3′) linking the *XhoI* and *NheI* restriction enzyme sites. The ancestral and mutant‐type constructs were named pSPL3‐WT and c.1221A > G, respectively. Two positive control mutants, c.1207‐1 G > A and c.1207‐26 A > G, that have been verified in previous reports were prepared using Quik Change™ mutagenesis kit (Stratagene, La Jolla, CA). All constructs were verified by direct sequencing.

Human embryonal kidney 293 T (HEK 293 T) and Hela cells were cultured in 5% CO_2_ incubator in DMEM supplemented with 10% fetal bovine serum and 1% penicillin–streptomycin (Invitrogen, CA, USA). One day before transfection, cells were plated onto 6‐well culture plate to grow to approximately 70% to 80% confluence in an antibiotic‐free medium. Cells were then transfected with 4 μg plasmid DNA (empty pSPL3 control, WT, c.1221A > G, and two positive controls each) using Lipofectamine 3000 (Invitrogen, CA, USA) according to the manufacturer's instructions. At 48 h after transfection, cells were harvested and total RNA was extracted using TRIzol reagent (Invitrogen, CA, USA), and used for RT/PCR to confirm the splicing patterns. First‐strand cDNA was synthesized from 1ug total RNA by random‐primed reverse transcription using PrimeScript 1st Strand cDNA Synthesis Kit (TaKaRa, Japan). To evaluate the pattern of transcripts from the transfected minigenes, the following vector‐specific primers were used for PCR amplification: a forward primer SD6 (5′‐TCTGAGTCACCTGGACAACC‐3′) and a reverse primer SA2 (5′‐ATCTCAGTGGTATTTGTGAGC‐3′). All transcripts were analyzed by sequencing.

### RNA analysis

cDNA was reverse‐transcribed from total RNA extracted from peripheral blood leukocytes. Splice mutations were detected by cDNA‐sequencing using one‐pair PCR primers spanning from exon 7 to 11 (forward: 5′‐CCTATTTGAGGGAGCAAGGTAA‐3′, reverse: 5′‐ATGTCTTGGTGCTGGTGGGAT‐3′; product, 464 bp).

## Results

### Clinical features of patients

As outlined in Table 1, four members (Ib, IIa, IIc, and IIIb) of the Chinese family were found to have hypertension, hyperkalemia, hyperchloremic metabolic acidosis, suppressed renin–angiotensin–aldosterone system, and normal renal function, which were in accordance with the clinical characteristics of PHAII, and abnormal findings on laboratory examinations and hypertension were promptly normalized by the administration of thiazides. Of note, the prominent feature of all these four patients were growth impairment, with short stature in three adults and failure to thrive in a child. Additionally, all three adult patients had a medical history of hypertension before the age of 18 years. All nonaffected individuals in this family demonstrated normal plasma potassium levels, normotension, and normal growth.

### Genetic analysis of patients

**Figure 2:**
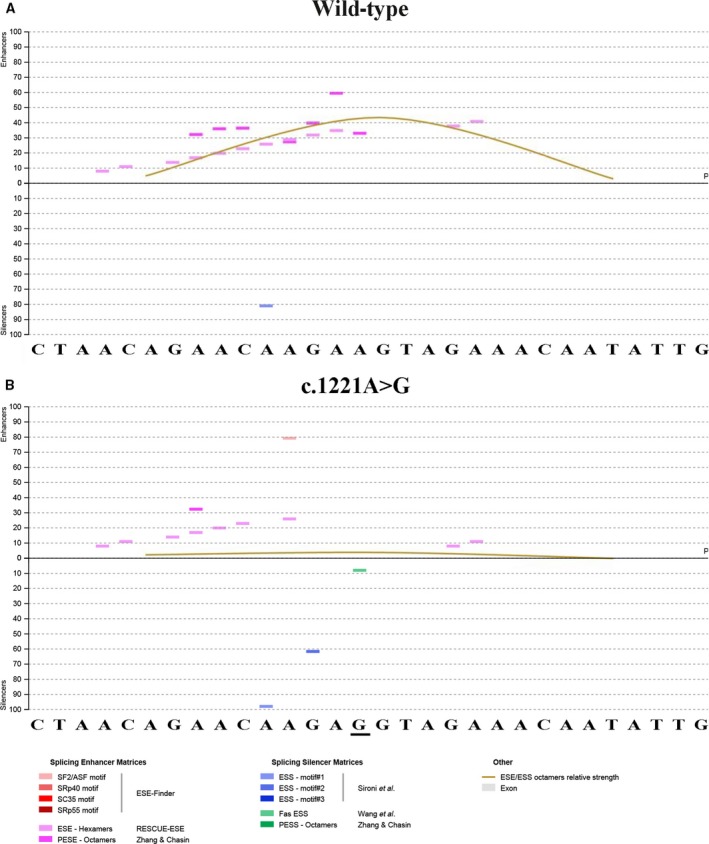
ESE and ESS motifs’ analysis of a 30‐nucleotide segment derived from the beginning of exon 9 containing WT (A) or mutant site (B) by Human Splicing Finder 3.0. Splicing enhancer matrices and splicing silencer matrices were displayed at the bottom, respectively.

*WNK1* and *WNK4* genes were preferentially screened in this study followed by detecting of *KLHL3* and *CUL3* genes. Sequencing analysis revealed no mutation in *WNK1*,* WNK4,* and *KLHL3*. In *CUL3*, a novel synonymous mutation c.1221A > G (p.Glu407Glu) located in exon 9 was found. This variation was only found in the four suffered patients while not in the other family members and 100 unrelated control subjects. *In silico* analysis by HSF3.0 software showed that dramatic changes of splicing regulatory motifs occur due to this mutation. As shown in Fig. 2, compared with wild‐type sequence, the mutation c.1221A > G led to a generation of several new putative ESSs such as ESS motif#1 (CAAGAGGT), ESS motif#2 (AGAGGTAG), and Fas‐ESS hexamers (CAAGAGGT) 22, 23, accompanying with a disruption of a variety of ESEs surrounding the mutation site 24, 25. Thus, we supposed a balance loss of splicing modulation and/or the splice site recognition between promotion and inhibition probably contribute to exon 9 skipping of *CUL3*.

### Functional analysis of the synonymous mutation

**Figure 3:**
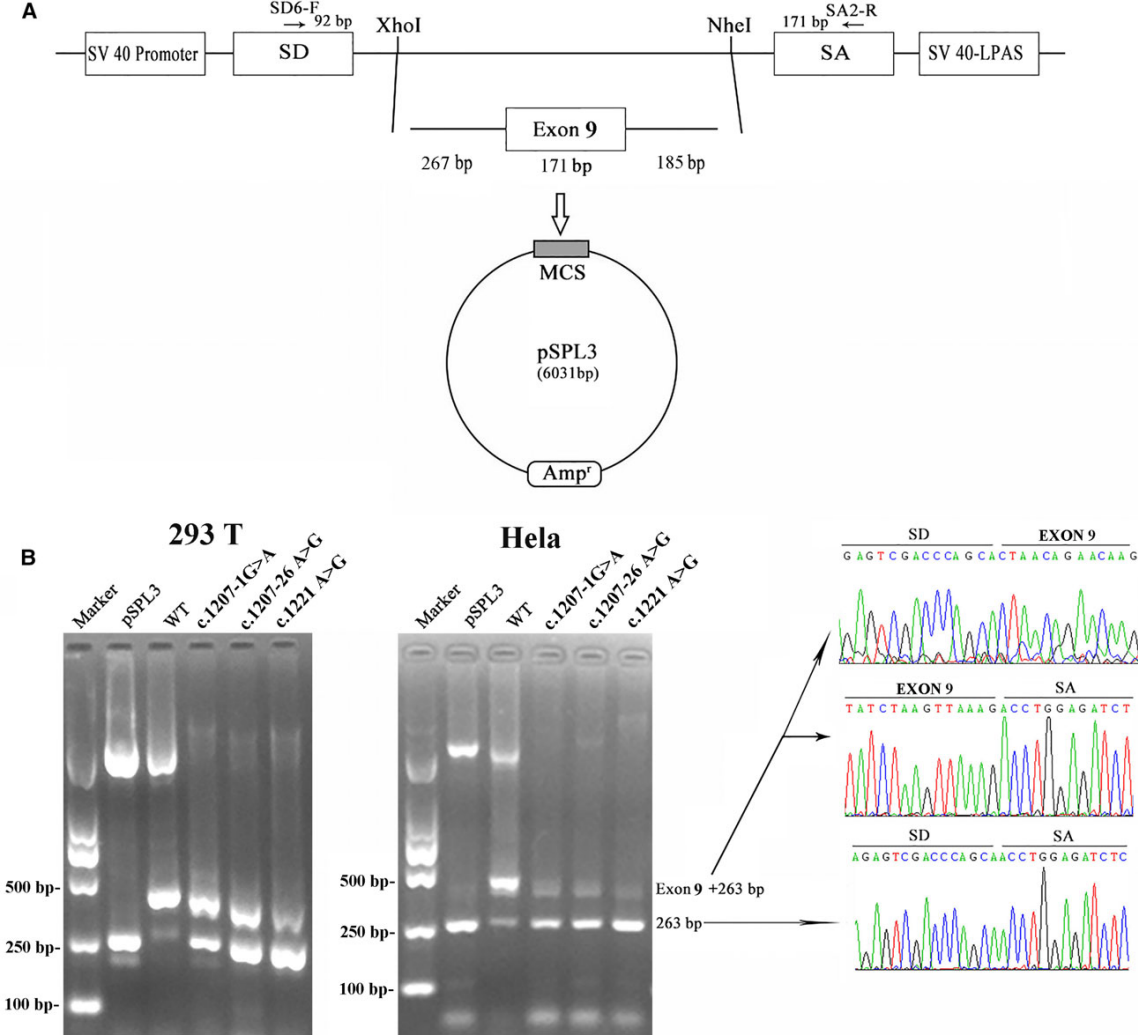
The minigene splicing assay based on the pSPL3 exon‐trapping vector. (A) The pSPL3 vector contains two exons SD and SA, and a functional intron, with transcription beginning following the SV40 promoter and ending at the LPAS (late poly (A) signal). Wild and c.1221A > G mutant CUL3 fragments containing 267 bp of intron 8, 171 bp of exon 9, and 185 bp of intron 9 were separately cloned into the XhoI and NheI cloning sites of the pSPL3 vector. (B) Agarose gel electrophoresis of RT/PCR products. SD6 and SA2 primers were designed for RT/PCR amplification of cDNA sequences generated by transfected 293T and Hela cells. Lane 1: marker; Lane 2: empty vector (263 bp); Lane 3–6 : 434 bp (263 bp + 171 bp) and 263 bp. MCS: multiple cloning sites.

To determine the effect of the synonymous c.1221A > G (p.Glu407Glu) on exon 9 splicing, exon trapping assay was performed as described above (Fig. 3A). The minigene assays showed that, in both HEK293T and Hela cells, the empty pSPL3 control and the c.1221A > G mutant constructs, as well as the two positive control mutants, all gave rise to a dominant 263‐bp PCR fragment missing exon 9 of *CUL3* gene, while the wild‐type yielded a main RT/PCR product of 434‐bp PCR fragments containing exon 9, and a minor product corresponding to 263‐bp fragments (Fig. 3B). Therefore, we determined that the synonymous mutation c.1221A > G (p.Glu407Glu) caused exon 9 skipping in the *CUL3* transcripts via a combination of *in silico* and *in vitro* assays.

### *In vivo* splicing verification

**Figure 4:**
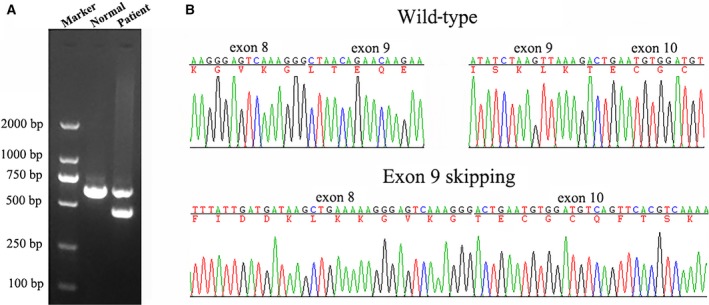
Electrophoresis for amplification and RNA sequence of CUL3 in a patient with pseudohypoaldosteronism type II and normal person. (A) Electrophoresis for amplification (B) upper panel, wild‐type electropherograms; lower panel, patient IVc electropherogram showing the absence of exon 9.

We then investigated whether the synonymous mutation c.1221A > G really led to exon 9 skipping in an actual patient from this family. The cDNA from the peripheral blood was amplified by PCR with a pair of primers spanning exon 7 to exon 11. By cDNA‐sequencing, the exon 9‐excluded transcript was identified only in the patient and not in a nonmutated individual (Fig. 4).

## Discussion

Aberrant splicing caused by a DNA mutation is a common pathogenic mechanism responsible for many genetic diseases, of which exon skipping is the predominant form 26. Previous evidence strongly indicates that all *CUL3* mutations associated with PHAII exclusively cause exon 9 skipping owning to affecting the correct splicing of exon 9, although the underlying mechanism may be still unclear 3.

It is well known that normal splicing process of pre‐mRNA is complex and elaborately regulated in humans. A series of splicing regulatory elements distributed in the whole genome control the correct splicing of the pre‐mRNA into mature mRNA. Alteration of these regulatory elements such as loss of a constitutive splice site, creation of a cryptic splice site, and alteration of a *cis*‐regulatory element may lead to aberrant splicing to cause disease 27, 28, 29.

In this study, we identified a novel synonymous mutation c.1221A > G (p.Glu407Glu) in four patients from a Chinese family, and sound evidence from *in vivo* and *in vitro* splicing assays confirmed this variant led to abnormal skipping of exon 9 as did other *CUL3* mutations associated with PHAII. Further *in silico* analysis by HSF3.0 demonstrated that mechanisms of the abnormal splicing due to this mutation might be related to alteration of various regulatory motifs. Usually, a series of juxtaposed sequence motifs such as ESEs and ESSs act in a combinatorial manner to regulate exon usage. They enhance or silence the use of adjacent splice sites by recruiting different protein factors 20. As shown in Fig. 2, this single exonic nucleotide change not only brings about destruction of many kinds of ESEs, but also creates a variety of ESSs, leading to a prominent decrease in the ratio of numbers of ESEs to ESSs; thus, the total strength of recognition and usage of adjacent splice site markedly reduced. We cannot help asking why a single base change altered so many predicted ESEs and ESSs? *In silico* analysis by HSF 3.0 revealed this might be related to the site of this mutation which is just localized in a high‐density region (+ 1 ~ +40 bp of exon 9) of ESEs (Fig. S1). Of note, besides this mutation c.1221A > G identified in our study, other two exonic variants c.1236G > A and c.1238A > G, which have been reported to be associated with PHAII, were also located in this region. Thus, that might be exactly why these mutations were recurrently found in this region. In addition, it should be noticed that intron 8 has a weak 3′ acceptor site (score 0.51, evaluated by BDGP, Fig. S2). It could be presumed that the exon–intron boundaries of exon 9 may not be correctly recognized without the assistance of the ESEs in the context of weak splice site. As a result, any changes involved the pivotal cis‐regulatory elements (ESEs or ESSs) in this region may lead to abnormal splicing of exon 9 although these motifs are not the core splicing signals such as 5'ss, 3'ss, and branch point sequence.

Particularly, this finding also implies a therapeutic approach that normalizes such aberrant splicing through the administration of specific reagents may apply to patients with PHAII who have these mutations. Nowadays, various types of antisense‐mediated modulation of splicing including correction of cryptic splicing, switching between alternative splice forms, exon inclusion, and reading frame correction have many applications 30, 31. Of all therapeutic applications, antisense‐mediated exon skipping for Duchenne muscular dystrophy (DMD) is closest to clinical application 32. As for our patients, using antisense oligonucleotides (AONs) which target ESSs induced by this mutation c.1221A > G to induce exon 9 inclusion might be one of potential treatment options worth trying. Cultured skin fibroblasts of these patients would be ideal cells to verify this supposition. Next step, we will carry out researches in this way. On the other hand, the contribution of each motif could not be distinguished clearly. Hence, the definite function of these splicing regulatory elements is needed to be further investigated in future study.

Regarding the phenotype, in agreement with previous reports and most subjects with mutations in *CUL3*, these patients in the study were diagnosed with evident hyperkalemic metabolic acidosis, growth impairment, and hypertension before age of 18 years, a more severe phenotype than those with mutations in *KLHL3*,* WNK1*, or *WNK4*. However, contrary to the finding that the majority of *CUL3* mutations in PHAII patients were *de novo*, this novel mutation c.1221A > G was transmitted as an autosomal dominant mode of inheritance in this family, a suggestion of no impairment of reproductive fitness.

In summary, in this study, we identified a novel exonic synonymous mutation c.1221A > G (p.Glu407Glu) in *CUL3*, which caused exon 9 skipping that confirmed by *in vivo* and *in vitro* splicing assays, in four patients from a Chinese kindred with PHAII. The potential mechanism of this mutation might be alteration of the balance of positive and negative splicing regulatory elements such as ESEs and ESSs. Our study provided more evidence for demonstrating the important role of exon splicing regulatory elements in pathogenesis of human genetic disease.

## Author contributions

XZ conceived and designed the experiments. LS and JL performed the experiments. LC and YL collected and performed the data analyses. LS wrote the manuscript, and IB and XZ revised the manuscript. All authors have reviewed the final manuscript and approved submitting for publication.

## Supporting information


**Fig. S1.** Schematic diagram of CUL3 exons with the scores of the acceptor/donor splicing sites (evaluated by BDGP software) illustrated above each exon.
**Fig. S2.** ESE and ESS motifs analysis of the whole exon 9 sequence by Human Splicing Finder 3.0, which flanked by 100 nucleotides upstream and downstream intronic sequences, respectively.
**Table S1.** A summary of CUL3 exon 9 mutations associated with PHA II.
