# Supplementary material for: A novel mutation in exon 9 of Cullin 3 gene contributes to aberrant splicing in pseudohypoaldosteronism type II
Source: FEBS Open Bio. 2018 Feb 10;8(3):461–9. doi: 10.1002/2211-5463.12389 (PMC5832971; doi:10.1002/2211-5463.12389)
Supplement: Supplementary file 1 — Fig. S1. Schematic diagram of CUL3 exons with the scores of the acceptor/donor splicing sites (evaluated by BDGP software) illustrated above each exon. Fig. S2. ESE and ESS motifs analysis of the whole exon 9 sequence by Human Splicing Finder 3.0, which flanked by 100 nucleotides upstream and downstream intronic sequences, respectively. Table S1. A summary of CUL3 exon 9 mutations associated with PHA II. [file FEB4-8-461-s001.doc]

Table S1. A summary of CUL3 exon 9 mutations associated with PHA II

| **Mutation** | **Clinical significance** | | **Reference** | |
| --- | --- | --- | --- | --- |
| 1. c.1207-28T>G | | Pathogenic | | Boyden et al. (2012) |
| 1. c.1207-26A>G | | Pathogenic | | Boyden et al. (2012) |
| 1. c.1207-12T>G | | Pathogenic | | Boyden et al. (2012) |
| 1. c.1207–6 T>G | | Pathogenic | | Osawa et al. (2013) |
| 1. c.1207-5T>A | | Pathogenic | | Boyden et al. (2012) |
| 1. c.1207-3C>T | | Pathogenic | | Boyden et al. (2012) |
| 1. c.1207-1G>A | | Pathogenic | | Boyden et al. (2012) |
| 1. c.1221A>G,p.Glu407Glu | | Pathogenic | | novel |
| 1. c.1236G>A (p.Leu412Leu) | | Pathogenic | | single submitter in ClinVar |
| 1. c.1238A>G (p.Asp413Gly) | | Pathogenic | | Boyden et al. (2012) |
| 1. c.1376A>G (p.Lys459Arg) | | Pathogenic | | single submitter in ClinVar |
| 1. c.1376_1377insG(p.Thr460Aspfs) | | Pathogenic | | single submitter in ClinVar |
| 1. c.1377G>A (p.Lys459Lys) | | Likely pathogenic | | single submitter in ClinVar |
| 1. c.1377G>C(p.Lys459Asn) | | Pathogenic | | Tsuji et al. (2013) |
| 1. c.1377+1G>C | | Pathogenic | | single submitter in ClinVar |
| 1. c.1377+3A>G | | Pathogenic | | single submitter in ClinVar |
| 1. c.1376_1377+4delAGGTAA | | Pathogenic | | Boyden et al. (2012) |


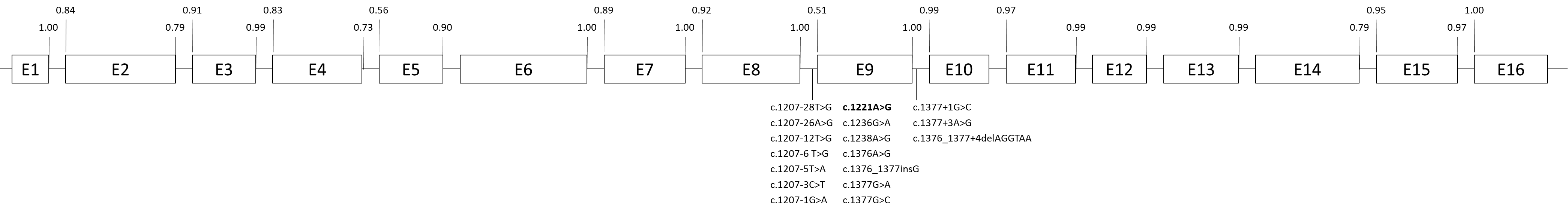


Fig S1. Schematic diagram of CUL3 exons with the scores of the acceptor/donor splicing sites (evaluated by BDGP software) illustrated above each exon. The already described *CUL3* pathogenic mutations as well as the one here described (the one in bold) are shown under the exon 9. Exons are in scale, introns are not in scale.


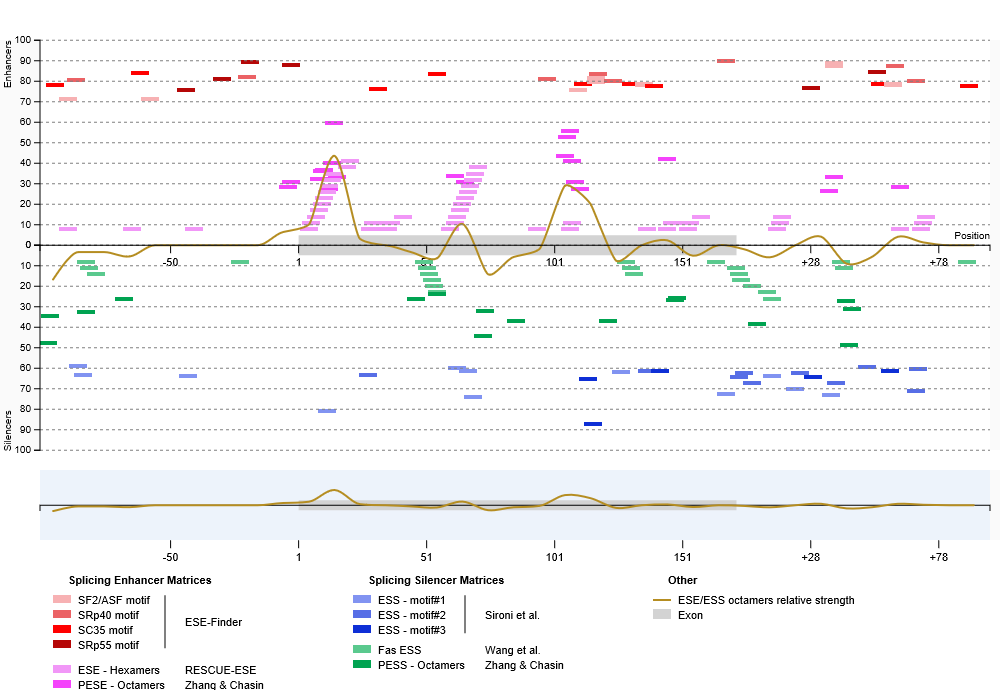


Fig S2. ESE and ESS motifs analysis of the whole exon 9 sequence by Human Splicing Finder 3.0, which flanked by 100 nucleotides upstream and downstream intronic sequences, respectively. Enhancer Matrices and Splicing Silencer Matrices were displayed at the bottom, respectively.
